# The therapeutic alliance in blended versus face-to-face cognitive behavioral therapy for adolescents and young adults with a depressive disorder

**DOI:** 10.1016/j.invent.2024.100776

**Published:** 2024-09-14

**Authors:** Miriam L.F.M. van Seters, Sanne P.A. Rasing, Mireille J. Huvenaars, Ad Vermulst, Denise H.M. Bodden, Yvonne A.J. Stikkelbroek

**Affiliations:** a GGZ Oost Brabant, Child and Adolescent Psychiatry, 5427, EM, Boekel, the Netherlands; b Behavioural Science Institute, Radboud University, 6500, HE, Nijmegen, the Netherlands; c Utrecht University, Clinical Child and Family Studies, 3508, TC, Utrecht, the Netherlands; d Altrecht, Child and Youth Psychiatry, 3524, SH, Utrecht, the Netherlands

**Keywords:** Adolescent and young adult depressive disorder, Blended cognitive behavioral therapy, Therapeutic alliance, Working alliance, Cognitive behavioral therapy

## Abstract

**Introduction:**

A depressive disorder during adolescence is a serious and disabling disorder, which has a high impact on the development of adolescents. Blended treatment, combining online and face-to-face sessions, is effective and can reduce some of the barriers for adolescents to use mental health care. There is a lack of knowledge about whether therapeutic alliance is established in blended treatment for adolescents and young adults suffering from a depressive disorder. This study examines whether the quality of the therapeutic alliance differs when cognitive behavior therapy (CBT) is delivered in combination with online intervention (b-CBT) compared to solely face-to-face (FtF-CBT) and the extent to which a stronger therapeutic alliance is associated with better treatment outcome.

**Methods:**

A pragmatic quasi-experimental design was used. Data collected within two separate studies were combined. A total of 85 participants (80 % female), aged 13–22 (mean = 16.63, SD = 1.92) were recruited within mental health care institutions and diagnosed with a depressive disorder (using K-SADS). Assessments were done at pre-treatment (T0), after five weeks (T1), after ten weeks (T2), post-treatment (T3) and one to four weeks after treatment (T4) and included measures of depressive symptomatology (CDI-2). The therapeutic alliance was measured at T1, T2 and T3 by the TASC. *t*-tests for independent samples were used to test differences in therapeutic alliance rates between b-CBT and FtF-CBT at post-treatment. A linear growth model for depressive symptoms based on five time points with Latent Growth Curve Analysis (LGCA) was used to test whether the therapeutic alliance is associated with depressive symptoms.

**Results:**

No differences in therapeutic alliance between b-CBT and FtF-CBT were found on either client-rated or therapist-rated therapeutic alliance. For both intervention groups, no significant association between the therapeutic alliance and depressive outcome was found.

**Discussion:**

This study shows that providing part of CBT using an online environment does not have a negative impact on the therapeutic alliance. In contrast to earlier research, no association was found between the therapeutic alliance and therapy outcome in neither the b-CBT nor the FtF-CBT intervention.

## Highlights

• No differences were found in the therapeutic alliance between blended and face-to-face CGT for adolescent depressive disorders

## 1 Introduction

Adolescents have a high risk of developing a depressive disorder (Shorey et al., 2022). Depression is associated with a high comorbidity such as substance abuse (Wilkinson et al., 2016), high suicidal risk (Portzky and Van Heeringen, 2009), poor academic performance and social functioning (Verboom et al., 2014) and a high risk of recurrence (Birmaher and Brent, 2007) making effective treatment in the adolescent phase of great importance. Studies on cognitive behavioral therapy (CBT) for adolescents with a depressive disorder show that treatment proves effective (Oud et al., 2019), though the outcomes are relatively modest (Zhou et al., 2015) with 60 % of the adolescents not responding to psychotherapy (Cuijpers et al., 2023) and a high drop-out rate (57 %) (Stikkelbroek, 2016). Hence, this substantiates the need for further adjustments of CBT targeting depressive disorders specifically for adolescents (Cuijpers et al., 2023; Oud et al., 2019). Blended treatment seems promising as a treatment for adolescent depressive disorders. Blended refers to a combination of an online part and a face-to-face part and has yet no clear definition making the blended formats differ from each other (the online part may be guided or unguided, the face-to-face and online part may be mixed or sequential).

Blended treatment is effective in targeting adolescents with depression (Rasing et al., 2021; Kobak et al., 2015; Sethi, 2013). It has several benefits that can help adolescents overcome practical barriers to seek help such as flexibility in time and location and reduction of transport costs (Gulliver et al., 2010; Radez et al., 2021). Blended treatment can also meet the developmental need of self-reliance in adolescence (Cerga-Pashoja et al., 2020). Little is known about the role of the therapeutic alliance within blended treatment for adolescents and young adults. Therapeutic alliance is considered an important factor in the effectiveness of therapy (Lambert and Barley, 2001).

In general, the pan theoretical version of therapeutic alliance is defined as one general factor with three different components; the mutual emotional connection (*the bond*) between the client and therapist, their agreement on the goals of therapy (*the goal*), and the tasks to be performed (*the task*) (Bordin, 1979). Within regular face-to-face CBT (FtF-CBT), the association between a stronger therapeutic alliance and a better treatment outcome is a consistent finding in adolescent therapy as well as adult therapy (Bose et al., 2022; Flückiger et al., 2018; Horvath et al., 2011; Karver et al., 2018; McLeod, 2011; Shirk and Karver, 2003) and is also confirmed in internet based interventions (Probst et al., 2022). In general, among predictors of treatment outcome (e.g. therapist competence or specific techniques), therapeutic alliance was the strongest predictor with small to moderate effects (Wampold and Imel, 2015). Young people are developmentally distinct from adults, and these differences inevitably influence the therapeutic alliance and its development criticizing the current definition of therapeutic alliance, which is based on adults and does not meet this requirements (Cirasola and Midgley, 2023). Adolescents have a developmental need for autonomy and self-reliance, which may complicate the forming of an emotional connection with an adult therapist (DiGiuseppe et al., 1996). Developing an agreement on goals (as part of the therapeutic alliance) may be particularly challenging since adolescents rarely seek help themselves; may have been referred to treatment by others; and/or lack self-awareness of their problems (DiGiuseppe et al., 1996; Gulliver et al., 2010). Specifically for the online part of b-CBT, it's assumed that the therapeutic alliance may be different in adolescents compared to adults, because adolescents are considered ‘digital natives’ who are often comfortable communicating and building relationships in a text-based online format (Mortimer et al., 2022). These differences stress the importance of research on the therapeutic alliance in adolescent therapy, more specific for blended treatment where research on the therapeutic alliance is scarce.

There is limited research investigating the difference in quality of the therapeutic alliance between blended and face-to-face treatment. For example, Ly et al. (2015) found that the therapeutic alliance was equally strong in a blended treatment for adolescents with depression compared to a face-to-face protocol. These findings are in line with findings on the therapeutic alliance in blended versus face-to-face treatment for adults (Askjer and Mathiasen, 2021; Kooistra et al., 2020). However, in a pilot study, Kobak et al. (2015) found that the therapist-rated alliance was significantly stronger in b-CBT compared to (face-to-face) treatment as usual for adolescents with depressive disorders. Kobak and colleagues suggested that use of online modules in combination with face-to-face contacts improved the therapeutic bond by helping the adolescent to learn the CBT concepts and skills, and helped to foster client engagement between the sessions, making therapy ubiquitous between sessions. Cerga-Pashoja et al. (2020) subsequently agreed that a blended format offers opportunities for reflection outside the therapist's office, as well as aiding memory and homework. In addition, the online part can be valuable for preventing therapeutic drift through tracking and bridging therapy content and progress from one session to another.

To learn more about the relationship between therapeutic alliance and outcome, collecting information from both the client and the therapist is important as it reflects the dyadic nature of their relationship. To our knowledge, research on the *alliance-outcome* association in b-CBT for adolescents coping with depression is not available. Regarding alliance-outcome association in b-CBT for adults, some studies found that therapist-rated alliance was predictive of the depressive outcome; whereas the client-rated alliance was not (Askjer and Mathiasen, 2021; Vernmark et al., 2019); other studies report no association between therapeutic alliance and outcome at all (Kooistra et al., 2020). These findings are in contrast with findings in regular FtF-CBT where both client and therapist rated alliance are related to outcome. Vernmark et al. (2019) found that only therapist-rated alliance is associated with outcome and suggested this may be specific for b-CBT. As in b-CBT the therapist rated the alliance only on the face-to-face part, whereas the participants rated their alliance scores on both the digital part and the face-to-face part.

Looking at adolescent alliance literature in general, an interesting finding is that in face-to-face therapy, adolescent-rated alliance is stronger related to treatment outcome than therapist-rated alliance (Shirk et al., 2008). Especially, the adolescent-reported alliance measured early in treatment predicted a decrease in depressive symptoms (Shirk et al., 2008; Gergov et al., 2021). Furthermore, Gergov et al. (2021) found that the discrepancy between the therapist and adolescent ratings was a significant predictor of undesirable treatment outcome. However, still little is known about the role of the therapeutic alliance in relation to outcome in blended treatment for adolescents with a depressive disorder.

The aim of this study was to examine the therapeutic alliance in b-CBT compared to FtF-CBT, and to address the following questions: (1) Is there a difference in therapeutic alliance between b-CBT and FtF-CBT as rated by clients as well as their therapists? We expect no difference in the therapeutic alliance between b-CBT and FtF-CBT, as rated by clients as well as therapists. (2) Is a therapeutic alliance as rated by adolescents and therapists related to outcome in b-CBT and FtF-CBT? We expect that a stronger therapeutic alliance is related to better outcome in both intervention groups.

## 2 Method

### 2.1 Study design

Data was collected within a pragmatic quasi-experimental design. Data had previously been collected in a RCT that compared FtF-CBT to treatment as usual (TAU) (collected December 2011 until June 2014) and data was collected in a later study on b-CBT intervention (collected November 2017 until December 2019). The research design of the first RCT is described in Stikkelbroek et al. (2013) and more details of the study on b-CBT can be found in Rasing, Stikkelbroek and Bodden, 2019a, Rasing et al., 2019b. The recruitment, eligibility criteria and assessments are identical in both studies. The samples were not significantly different with respect to gender, ethnicity, education level (tested with Fisher's exact test with *p* = 0.593, 0.671 and 1.000 respectively), age and severity of depression (tested with *t*-tests for independent samples with t(83) = 0.41, *p* = 0.683 and t (64) = −0.30, *p* = 0.765 respectively) at the start of the intervention. In the RCT, participants were randomly assigned to the intervention condition (FtF-CBT) or the control condition (TAU). In the study on b-CBT, all participants were recruited and assigned to blended CBT.

Both studies have been approved by the Medical Research Committee (METC Utrecht) (protocol NL61804.041.17 approved on October 10, 2017 and protocol NL34064.041.10 approved on June 14, 2011) and were registered in the Dutch Trail Register (NTR; Trail Ids: NTR 6759 registered on October 16, 2017 and registered NTR2676 on January 3, 2011).

### 2.2 Participants & procedure

Participants were adolescents between the ages of 12 and 21 who suffered from a major depressive disorder or persistent depressive disorder and who had been referred to a mental health institution for treatment, which were similar in both samples. Two adolescents turned 22 shortly after they gave consent and remained included.

The presence of the diagnoses of a depressive disorder was measured by the K-SADS-PL, (Kiddie Schedule for Affective Disorders and Schizophrenia for School-Age Children - Present and Lifetime Version) is a semi-structured diagnostic interview used to assess psychiatric disorders in children and adolescents, both in the present and the past (Kaufman et al., 1997). The interview was conducted by a trained and independent research assistant. Enrollment occurred after written consent was obtained from the adolescent and in case the adolescent was under the age of 16 the parents had to give written consent as well. Exclusion criteria for the adolescent were: an acute suicide risk, a drug abuse disorder (as primary disorder), an autistic spectrum disorder (as primary disorder) or a bipolar disorder (as primary disorder), as measured by the K-SADS-PL. Participants who were referred to intensive treatment such as day care or hospitalization, or had insufficient knowledge of the Dutch language were also excluded. The initial sample size after combing the two samples was 85 participants (b-CBT *n* = 41, FtF-CBT *n* = 44). Participants were registered by their therapist as dropout when they stopped the intervention early, they could not be reached by the therapists, when a referral to more intensive treatment was necessary due to an increase in symptoms, or when participants stopped the intervention prematurely because they experienced sufficient improvement.

In the b-CBT study, 19 therapists were involved, and in the FtF-CBT study, there were 37 therapists providing treatment, which makes a total of 56 therapists involved in this study. Most therapists (95.0 %) were licensed psychologists MSc with one or more clinical registrations, CBT trained (three were in training to become CBT therapist) and had at least one year of experience within professional mental health. Prior to the study, the therapists followed a two-day training in the FtF-CBT “Doepressie protocol”. In the b-CBT an extra training day was added on how to provide the blended protocol.

Demographic data were collected from the start of the study. Assessment points were pre-treatment (T0), five weeks after the start of the intervention (T1), ten weeks after the start (T2), posttreatment (one to four weeks after the end of treatment) (T3) and at the six-month follow-up (T4). Participants and therapists were asked to score the therapeutic alliance scale at T1, T2 and T3, so only these assessment points are used.

### 2.3 Interventions

The FtF-CBT is a protocolized individual cognitive behavioral therapy intervention (Doepressie intervention www.doepressie.nl), which is based on the evidence-based program ‘Coping with Depression Course for Adolescents’ (CDW-A). The intervention consists of 15 weekly sessions with a duration of 45 min and mainly uses cognitive-behavioral principles. Parents received two face-to-face sessions in which they received psychoeducation.

The b-CBT is an adaptation of the FtF-CBT in which the fifteen sessions have been adapted into four online modules. A minimum of five face-to-face sessions is required. One face-to-face session is scheduled at the start of each online module and one at the end of the treatment. In addition, up to ten face-to-face sessions can be added on request of the participant (maximum 15 total). In between, the participant can contact the therapist online at any time. The online program consists of a number of pages where a page is registered as completed when it has been opened by the client for a minimum amount of time. At the same time the therapist receives an automatic notice when the participant has completed a task. This blended version remains unchanged in terms of content compared to the FtF-CBT protocol. Parents received two face-to-face sessions in which they receive psychoeducation.

### 2.4 Outcome measures

Depressive symptoms were measured with the Child Depression Inventory-2 (CDI-2; Bodden et al., 2016); a self-report questionnaire for children (8 to 21 years) that reflects affective, behavioral, and cognitive symptoms of a depressive disorder. The CDI-2 version consists of 28 questions, each with three response options: non-depressed (value 0; “I am sometimes sad”); mildly depressed (value 1; “I am often sad”), and clearly depressed (value 2; “I am always sad”). The total score on the CDI-2 is converted into a percentile score or compared to a cut-off score. Previous research on the psychometric properties of the total score has shown moderate to adequate outcomes in a general population, with test-retest reliability (*r* = 0.60) and convergent validity (*r* = 0.77). Cronbach's alpha is 0.89 for 13–16 year olds and 0.85 for 17–21 year olds (Bodden et al., 2016).

The therapeutic alliance was measured with the Dutch version of the Therapeutic Alliance Scale for Children which is uniquely designed for children and adolescents (Shirk and Saiz, 1992) and translated in Dutch for research purposes. The TASC uses the concept of alliance according to Bordin (Bordin, 1979; Elvins and Green, 2008). Both clients and therapists filled out the TASC, a twelve item self-report questionnaire with a four point Likert scale (for example: “I liked spending time with my therapist”; Not at all - A little - Mostly - Very much). A total sum score can be calculated (range from 12 to 46), as well as two subscale scores on Affective bond (for example: “I enjoy spending time with my therapist”) and Collaboration on Task & Goal (for example “I think my therapist and I work well together on my problems”). Within this study Cronbach's alpha are 0.85, 0.81 and 0.66 (total sum score, affective bond and collaboration respectively) for the adolescent version and 0.74, 0.73 and 0.76 respectively for the therapist version.

### 2.5 Statistical analysis

To test differences between dropouts and completers in age and depressive symptoms at start of the intervention, we used the *t*-test for independent samples. For educational level, sex, ethnicity and proportions dropout Fisher's exact test was used. Due to the pragmatic study design, no prior power calculations could be performed. The power of this study (*N* = 85) for two-tailed independent sample *t*-tests turned out to be 0.62 with a type I error of 0.05 and a medium effect size of 0.5 (Cohen's *d*).

**Fig. 1:**
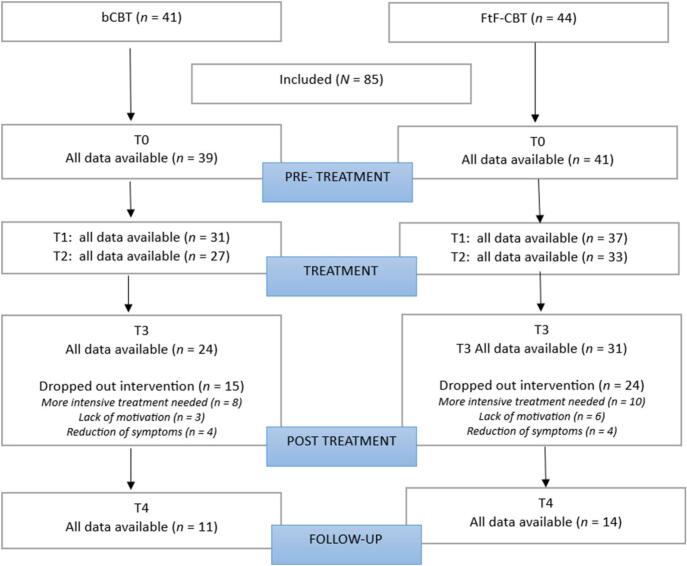
Flow chart of participants.

For the first research question, t-tests for independent samples was used to test differences between b-CBT and FtF-CBT with respect to the three therapeutic alliance variables. To test whether therapeutic alliance has an effect on depressive symptoms within the b-CBT-group and the FtF-CBT group, we first tested a linear growth model for depressive symptoms based on five time points (T0, T1 (5 weeks after start treatment), T2 (10 weeks after start treatment), T3 (4 months after start treatment) and T4 (6 months after start treatment) with Latent Growth Curve Analysis (LGCA) using the statistical package Mplus version 7.2 (Muthén and Muthén, 1998-2015). We used the Full Information Maximum Likelihood (FIML) estimator to handle missing data under the assumption that missing data are Missing At Random (Enders, 2010). Each participant with at least one score on depressive symptoms over time is included in the analyses. Two growth parameters were estimated: the intercept i (starting value of depressive symptoms at start of intervention) and the slope s (representing the degree to which depressive symptoms decrease per month). The fit measures of the growth model are Chi-square (df) with *p*-value (if *p* > 0.05, the fit is good), the Comparative Fit Index (CFI) (values >0.95 indicating a good fit and values >0.90 an acceptable fit) and Root Mean Squared Error (RMSEA) (values <0.05 indicating a good fit and values <0.08 an acceptable fit) (Kaplan, 2000; Kline, 2010). Monte Carlo power analysis according to Muthén and Muthén (2002) with five time points, medium effect size, Type I error of 0.05, sample sizes of 41 and 45 and dropout proportions at each time point calculated from Fig. 1 showed that the power to find significant slopes is 0.76 for the b-CBT group and 0.80 for the FtF-CBT group. The second step in the analysis was to test whether the expected decrease of depressive symptoms over time (the slope *s*) was affected by therapeutic alliance. Therapeutic alliance measured at T1, T2 and T3 was included in the latent growth curve model of depressive symptoms as time varying covariates. Depressive symptoms at T1, T2 and T3 was regressed on therapeutic alliance at T1, T2 and T3 respectively. It is expected that a higher score on therapeutic alliance is associated with an additional decrease in depressive symptoms resulting in a more negative slope (Bollen and Curran, 2006). After introducing the three therapeutic alliance variables T1-T3 in the growth model as covariates (with the result that depressive symptoms T1-T3 were corrected for the effects of therapeutic alliance T1-T3), the growth parameters were estimated again and the resulting slope compared with the slope of the initial model. Due to the small sample sizes, the LGCA was performed for each alliance subscale separately. Differences between the initial slope and the slopes with correction for the alliance variables was tested with paired *t*-tests assuming normal distributions of the sampling distribution of differences between the initial slope and the corrected slope. This assumption appeared to be correct because there were very minimal differences with the results of the bootstrap sample t-test (the latter with no assumption about the sampling distribution of differences).

## 3 Results

### 3.1 Dropouts

In the b-CBT condition, 15 of the 41 (36.6 %) dropped out of the intervention. In the FtF-CBT condition 24 of the 44 (54 %) dropped out of the intervention. In total 39 of the 85 participants (46 %) did not complete the intervention. The proportions of dropout of b-CBT and FtF-CBT were not significantly different (*p* = 0.128). Also, no significant differences were found between completers and drop-outs for age (t (83) = 1.00, *p* = 0.321), depressive symptoms at T0 (t (73) = −0.50, *p* = 0.619), educational level (*p* = 0.814), sex (*p* = 1.000) and ethnicity (*p* = 1.000). An overview of the participants in this study is shown in Fig. 1.

### 3.2 Demographics

A total of 85 participants (b-CBT *n* = 41, FtF-CBT *n* = 44), aged 13 to 22 years (*M* = 16.63, *SD* = 1.92) participated in the study. 80.0 % of the participants were female, 3.2 % of the participants had completed primary education, 40.3 % of participants completed secondary education and 56.5 % of participants completed higher education. Most participants had a Dutch background (96.9 %).

During the intervention and the follow-up period, 5 serious adverse events occurred. In the b-CBT intervention, two participants attempted suicide for which they were hospitalized and therefore the participation in the study was terminated. In the FtF-CBT intervention, one participant attempted suicide during the study and dropped out as more intensive treatment was indicated, one participant attempted suicide after completion of the study and one participant died after suicide in the follow-up period between six and twelve months after the intervention.

### 3.3 Treatment intensity

In the FtF-CBT, the participants completed the intervention on an average of 15 sessions (mean = 15.06, *SD* = 4.05, range 6 to 27 sessions), which is in line with the protocolized 15 sessions. In the blended intervention, the participants followed between 0 and 12 face-to-face sessions (mean = 5.77, *SD* = 3.22). The b-CBT and FtF-CBT interventions differed by definition in the degree of face-to-face treatment intensity. In addition, the participants who completed the b-CBT on average completed 57.6 % (range 0–98.7 %) of the online program.

### 3.4 Differences in therapeutic alliance between b-CBT and FtF-CBT

The results of the *t*-tests for possible differences in therapeutic alliance between the FtF-CBT and the b-CBT groups are shown in Table 1. Neither the total alliance scale nor the two subscales showed significant differences between the b-CBT and FtF-CBT groups on T1, T2 and T3 for both participant-rated and therapist-rated alliance, all *p*-values are >0.05.

**Table 1:** Means, SD's and t-tests for differences in therapeutic alliance between FtF-CBT and b-CBT.

		Participant						Therapist					
		b-CBT *n* = 41		FtF-CBT *n* = 44				b-CBT *n* = 41		FtF-CBT *n* = 44			
		*M*	*SD*	*M*	*SD*	t	*p*	*M*	*SD*	*M*	*SD*	t	*p*
T1	total	36.27	6.96	36.62	5.22	0.20	0.847	34.90	4.50	34.61	5.63	−0.22	0.825
	affective bond	17.64	4.04	17.46	3.04	−0.17	0.865	16.59	2.35	16.12	2.93	−0.68	0.498
	collaboration	18.64	3.14	19.15	3.00	0.58	0.563	18.31	2.78	18.48	3.06	0.23	0.816

T2	total	35.00	5.27	36.39	4.98	0.83	0.411	34.48	5.14	35.95	6.16	0.83	0.410
	affective bond	17.20	2.73	17.22	3.12	0.02	0.981	16.19	2.48	16.85	3.15	0.75	0.460
	collaboration	17.80	3.22	19.17	3.02	1.35	0.187	18.29	3.15	19.10	3.45	0.79	0.434

T3	total	36.25	6.62	35.90	6.02	−0.17	0.869	35.32	5.48	34.59	7.27	−0.40	0.695
	affective bond	17.06	3.36	17.52	3.14	0.43	0.670	16.77	2.83	16.55	3.84	−0.23	0.820
	collaboration	19.19	3.53	18.38	3.56	−0.69	0.497	18.55	3.00	18.03	3.89	−0.51	0.612

### 3.5 Association between therapeutic alliance and depressive symptoms within b-CBT and FtF-CBT

To test the effect of therapeutic alliance on depressive symptoms within both groups, we first tested a growth model for depressive symptoms over time (with five time points). The fit of the linear growth model was good with Chi-square (10) = 10.73, *p* = 0.379, CFI = 0.989 and RMSEA = 0.043 for the b-CBT-group and less acceptable for the FtF-CBT-group with Chisquare (10) = 21.98, *p* = 0.015, CFI = 0.822 and RMSEA = 0.175. Despite this, the latest model will be accepted. For small samples poor global fit indices like CFI and RMSEA can be misleading: they may still be consistent with a good approximation of individual growth curves (Coffman and Millsap, 2006). Graphical examination of the linear approximation supported this. The estimated mean intercept i was 26.18 (estimated starting value at T0) with mean slope s = −1.40 (*p* = 0.000) for the b-CBT-group and *i* = 24.80 and s = −2.16 for the FtF-CBT-group, the mean slopes indicating that depressive symptoms decreased monthly with 1.40 and 2.16 units respectively. For each participant with at least one observation on depressive symptoms over time a growth curve with intercept and slope was estimated, resulting in 39 individual growth curves for both groups. The next step was regressing the therapeutic alliance variables at T1, T2 and T3 on depressive symptoms at T1, T2 and T3 respectively in the latent growth curve model of depressive symptoms for participants and therapists within the b-CBT and the FtF-CBT groups. The resulting mean slopes (all significant with *p* < 0.001) and test results are given in Table 2. Each of the corrected slopes are compared with the initial slope using paired *t*-tests. No significant effects were found of therapeutic alliance on depressive symptoms in the b-CBT group as well as in the FtF-CBT group.

**Table 2:** Initial mean slopes of depressive symptoms, corrected mean slopes for therapeutic alliance and results of paired t-tests for differences between initial mean slope and corrected mean slopes.

	b-CBT *n* = 41				FtF-CBT *n* = 44			
	slope s	*p*^1^^)^	t(38)^2^^)^	*p*^3^^)^	slope s	*p*^1^^)^	t(38)^2^^)^	*p*^3^^)^
Initial slope depressive symptoms	−1.40	0.000			−2.16	0.000		
Participants								
corrected for total score	−1.38	0.000	−0.86	0.392	−2.36	0.000	1.80	0.079
corrected for affective bond	−1.49	0.000	1.00	0.326	−2.38	0.000	1.28	0.209
corrected for collaboration	−1.37	0.000	−0.72	0.476	−2.19	0.000	0.28	0.783

Therapists								
corrected for total score	−1.32	0.003	−0.66	0.516	−2.22	0.000	0.50	0.618
corrected for affective bond	−1.35	0.001	−1.42	0.164	−2.04	0.000	−1.24	0.222
corrected for collaboration	−1.37	0.001	−0.44	0.661	−2.34	0.000	1.53	0.134

1 significance level of slopes.

2 t-value of paired *t*-tests with df = 38 for differences between initial slope and corrected slopes.

3 significance level of paired t-test.

## 4 Discussion

The first aim of this study was to examine whether the quality of the therapeutic alliance, as rated by adolescents and young adults diagnosed with a depressive disorder and their therapists, differs between b-CBT and FtF-CBT. The second aim was to examine whether a higher therapeutic alliance is related to lower depressive symptoms after b-CBT and FtF-CBT. Our results show that (1) no differences were found in adolescent-rated and therapist-rated alliance between b-CBT and FtF-CBT, neither on the overall score nor on the subscale scores *(affective bond & collaboration on task and goal*). (2) We did not find a significant association between a higher therapeutic alliance and the decrease of depressive symptoms in for both participant-rated as therapist-rated alliance in b-CBT and FtF-CBT.

First, our findings show no difference in the quality of the therapeutic alliance between b-CBT and FtF-CBT in adolescents, which are in line with previous findings that the therapeutic alliance ratings in a blended format for adolescents coping with depression are not different from therapy alliance ratings of a face-to-face format (Kobak et al., 2015; Ly et al., 2015). The results also correspond with findings in adults studies (Askjer and Mathiasen, 2021; Kooistra et al., 2020). Our results support the idea that replacing a part of the face-to-face therapeutic protocol into interactive online modules supplemented by face-to-face sessions ensure a similar level of therapeutic alliance as in regular face-to-face treatment; while the blended format in turn may be an alternative specifically suitable for the adolescent. Blended treatment can overcome the practical barriers that impede adolescents such as lack of time and transport (Gulliver et al., 2010; Radez et al., 2021), while at the same time it can meet the developmental needs of the adolescents such as the need for self-reliance (Cerga-Pashoja et al., 2020).

Despite the lack of differences in therapeutic alliance, it can be argued whether the way the therapeutic alliance is conceptualized and measured by the TASC questionnaire is compatible between both interventions. First, some authors suggested that since the definition of therapeutic alliance is based on face-to-face therapy, it is therefore not necessarily the same in a blended format. In blended treatment there is less variation on the goals and cooperation, which may have an effect on the way the therapeutic alliance is scored by participants (Berry et al., 2018). Explorative research found that when studying therapeutic alliance in online treatment for adults only, the three dimensions of Bordin (1979) are not sufficient. They propose an additional dimension namely ‘usability heuristics’, which refers to aspects related to the intrinsic properties of digital intervention programs, such as accessibility or aesthetic appeal, that favor the development of users' autonomy and their commitment to the therapeutic process (Doukani et al., 2020). Moreover, in the specific case of adolescents, the impact of technology on the therapeutic alliance may be different because of how adolescents are used to communicate in de digital world (Mortimer et al., 2022). This, in turn, places a demand on the therapist because the therapist must be able to understand the digital language, adolescents use to communicate with their therapists, which in turn may affect the way the therapeutic alliance is established (Pagnotta et al., 2018).

Second, our findings regarding therapeutic alliance and treatment outcome are partial in line with the current literature, in which no association between patient-rated and therapist-rated alliance scores in b-CBT and outcome were found (Kooistra et al., 2020). However other studies on blended CBT in adults with a depressive disorder have found that therapist-rated alliance was predictive of symptom reduction; as the client-rated alliance was not (Askjer and Mathiasen, 2021; Vernmark et al., 2019). In our study, the same explanation as put forward by Kooistra could be applied for both the b-CBT as the Ftf-CBT group. In our study as well as in Kooistra's study, high ratings of therapeutic alliance by both participants as well as therapists were found, thus restricting variance in this variable and limiting the feasibility of detecting associations between therapeutic alliance and change in depression severity. This so called ceiling effect is a well-known problem in research on therapeutic alliance and is considered an important argument why other studies could not find an alliance-outcome association (Bickman et al., 2012; McLeod, 2011).

### 4.1 Strengths and limitations

This study has several strengths. First, participants were recruited within routine mental health care and diagnosed with a depressive disorder meaning that findings can be generalized to clinical care. Second, by turning a CBT protocol directly into interactive online modules, without changing its content, a direct comparison of blended and face-to-face CBT was possible and it can be ruled out that differences between the two interventions stem from differences in the therapeutic protocol used. Third, in regard to the therapeutic alliance: both adolescents and therapists rated the alliance and thereby the reciprocal nature of the therapeutic alliance was addressed. The therapeutic alliance was also measured multiple times, whereas most research in youth only assessed the therapeutic alliance at one point, mostly at the end of the intervention. This complicates assessment of the direction of the relationship between alliances and outcome, that is, whether the alliance drives symptom improvement and it is not a product of it (Cirasola and Midgley, 2023).

There are also some limitations that need to be taken into account while interpreting these results. First, as a result of limited power due to the relatively small sample size it is hard to detect small to moderate differences. The relatively high drop-out (46 % of the 85 participants) further reduces power, making the chance of finding significant results small, especially for the first research question (differences in therapeutic alliances between b-CBT and FtF-CBT). For the second research question (a higher therapeutic alliance is related to lower depressive symptoms with 39 slopes in each group) the post hoc power turned out to be 0.86 (paired *t*-test, Type I error 0.05 two-sided, medium effect size). Moreover, the latent growth models have sufficient power to detect significant slopes. Second, due to the pragmatic study design which combines data form two different samples in the analysis, randomization of the participants was not possible. The two samples match each other on the most important parameters, but differ in the time at which the data was collected. Third, the treatment drop-out was high, although in line with other clinical samples (Weisz et al., 2006). Due to the small sample size and the high drop-out, it was not possible to look further into the relationship between the differences and similarities of the therapeutic alliance between the client and therapist perspectives and outcome. This is unfortunate because addressing the degree of alliance agreement or divergence in the adolescent-therapist dyad is recognized as an important factor to improve therapy. For example, Gergov et al. (2021) found that the level of discrepancy between the therapist and adolescent ratings was a significant predictor of undesirable treatment outcome.

### 4.2 Future research

Some recommendations for future research can be given. To date, limited research has been done on how the therapeutic alliance is conceptualized in blended treatment, specifically in the adolescent phase. As stated before, the way the therapeutic alliance is conceptualized is derived from adult literature and criticized in adolescent literature as it is not developmentally complex enough (such as the need for self-reliance) (Horvath, 2018). At the same time, research on the therapeutic alliance in blended conditions show that Bordin's conceptualization may be not sufficient and need to be conceptualized in a different way in blended interventions. Research has suggested that technology forms a third factor in the relationship between client and therapist and need to be addressed in the measurement of the therapeutic alliance (Vernmark et al., 2019). Making the development of new conceptualizations and measurement tools for the therapeutic alliance for blended interventions by adolescents of great importance. The last decade, some researchers have shifted their attention to rupture and repairments of the therapeutic alliance. Safran and Muran (2006) redefined Bordin's concept of alliance as a continuous, dynamic process of intersubjective negotiation between client and therapist, characterized by ruptures, moments of deterioration in its quality and repairments, and moments in which such tensions are resolved. The use of this more dynamic conceptualization of therapeutic alliance is considered of importance in understanding early drop-out (Cirasola and Midgley, 2023) and the association of drop-out with outcome. Eubanks et al. (2018) found for example that unresolved ruptures predicted poor therapy outcomes and higher dropout making this theme important for further research on the therapeutic alliance. Furthermore, the definition of blended treatment is still somewhat heterogeneous in literature. For example, sometimes an intervention therapy is described as blended when there is only email contact in addition to face-to-face contact. The differences in definitions between studies complicate direct comparison of studies, especially for research on therapeutic alliances, because therapist involvement in the internet part may differ in nature and synchronicity.

### 4.3 Clinical implications

An important clinical implication of this study is that results show that replacing a portion of the therapeutic protocol into interactive online modules supplemented by face-to-face sessions gives equivalent results in terms of quality of the therapeutic alliance. This knowledge may help therapists to overcome their doubts and contribute to a better implementation of blended care. At the same time, the partial online environment can help to overcome barriers for adolescents to seek help and may meet specific needs of the adolescent coping with depression.

## 5 Conclusions

To our knowledge, this study was the first to examine the association between the therapeutic alliance and outcome in blended treatment for adolescents with a depressive disorder. No differences in therapeutic alliance between b-CBT and FtF-CBT were found on either client-rated or therapist-rated therapeutic alliance. For both intervention groups, no significant association between the therapeutic alliance and depressive outcome was found. Possibly, ceiling effects restricting the variance in alliance scores could limit the feasibility of detecting associations between therapeutic alliance and depressive outcome.

## Authors' contributions

MS, YS, and SR conceptualized and contributed to the design of the current study. MS wrote all the sections in the manuscript. SR, MH and YS reviewed and revised all sections of the manuscript. AV assisted in the planning, execution of data analyses, and description of the results. All authors have made substantive intellectual contributions to the paper, read and approved the final manuscript.

## Declaration of competing interest

Yvonne Stikkelbroek translated Doepression face-to-face into Dutch and developed the blended version of the intervention, for which she receives no direct payments. The other authors of the current study declare no conflict of interest.
